# Frontal cortex function as derived from hierarchical predictive coding

**DOI:** 10.1038/s41598-018-21407-9

**Published:** 2018-03-01

**Authors:** William H. Alexander, Joshua W. Brown

**Affiliations:** 1 Ghent University, Ghent, Belgium; 2 Indiana University, Bloomington, USA

## Abstract

The frontal lobes are essential for human volition and goal-directed behavior, yet their function remains unclear. While various models have highlighted working memory, reinforcement learning, and cognitive control as key functions, a single framework for interpreting the range of effects observed in prefrontal cortex has yet to emerge. Here we show that a simple computational motif based on predictive coding can be stacked hierarchically to learn and perform arbitrarily complex goal-directed behavior. The resulting Hierarchical Error Representation (HER) model simulates a wide array of findings from fMRI, ERP, single-units, and neuropsychological studies of both lateral and medial prefrontal cortex. By reconceptualizing lateral prefrontal activity as anticipating prediction errors, the HER model provides a novel unifying account of prefrontal cortex function with broad implications for understanding the frontal cortex across multiple levels of description, from the level of single neurons to behavior.

## Introduction

The frontal lobes are central to volition and higher cognitive function, especially goal-directed behavior^1–3^. Recent work has highlighted reinforcement learning^4–6^, performance monitoring^7,8^, and hierarchical abstraction and working memory^9–11^ as key elements of frontal function, often under the framework of cognitive control^12^. Considering the range of methods and perspectives applied to investigating prefrontal cortex (PFC), there is a clear need for a common framework for interpreting the variety of functions assigned to the frontal lobes.

Within the past decade, predictive coding has emerged as just such a potentially unifying framework for understanding the organization and function of the brain^13^. Hierarchical predictive coding, as well as related approaches including free energy^14^ and Hierarchical Bayesian Inference^15^, generally treat bottom-up processing of information in the brain as a source of evidence that must be “explained away” by top-down processes carrying information regarding the likely causes of sensory information. In the predictive coding framework, top-down processes provide predictions from superior hierarchical levels to inferior levels, while residual prediction errors, i.e., input that cannot be accounted for by the predictions supplied by top-down processes, are carried from inferior levels to superior levels. This motif of top-down predictions and bottom-up prediction errors repeats through successive hierarchical iterations, forming a sophisticated processing stream composed of “dumb processes that correct… error in the multi-layered prediction of input”^13^. Predictive coding accounts have achieved great success in accounting for effects related to the processing of sensory input^16–22^. Given this success in accounting for the structure and function of the brain in early sensory areas, it has been suggested^13^ that the predictive coding framework might be extended to account for the organization of brain regions underlying sophisticated cognitive processes, especially the frontal lobes.

There are several reasons to believe that predictive coding formulations may indeed map well to PFC in addition to primary sensory areas. PFC is generally considered to be organized hierarchically along a rostrocaudal abstraction gradient^9,10,23,24^, with rostral regions coding for abstract rules and task sets, while caudal regions represent concrete stimulus-response associations. Significant portions of PFC are specialized for reporting error as a deviation from predicted events^7,25^, and distinct regions within medial PFC (mPFC) appear to encode error at different levels of abstraction^26,27^, while regions within dorsolateral PFC (dlPFC) appear to encode hierarchical task set information^23^ and to contextualize behavioral responses based on a learned model of the environment^10,24^. However, while convergent evidence suggests that predictive coding accounts of brain function and organization may indeed extend into the frontal lobes, this proposed extension has remained largely hypothetical, and significant outstanding questions remain to be answered. Among these questions is whether the predictive coding framework can be leveraged to capture high-level cognitive behaviors, generally understood to rely on the frontal lobes, as well as how a predictive coding account, based on the computation of progressively more abstract error information, might inform our understanding of the information represented by single neurons and regions in PFC.

**Figure 1:**
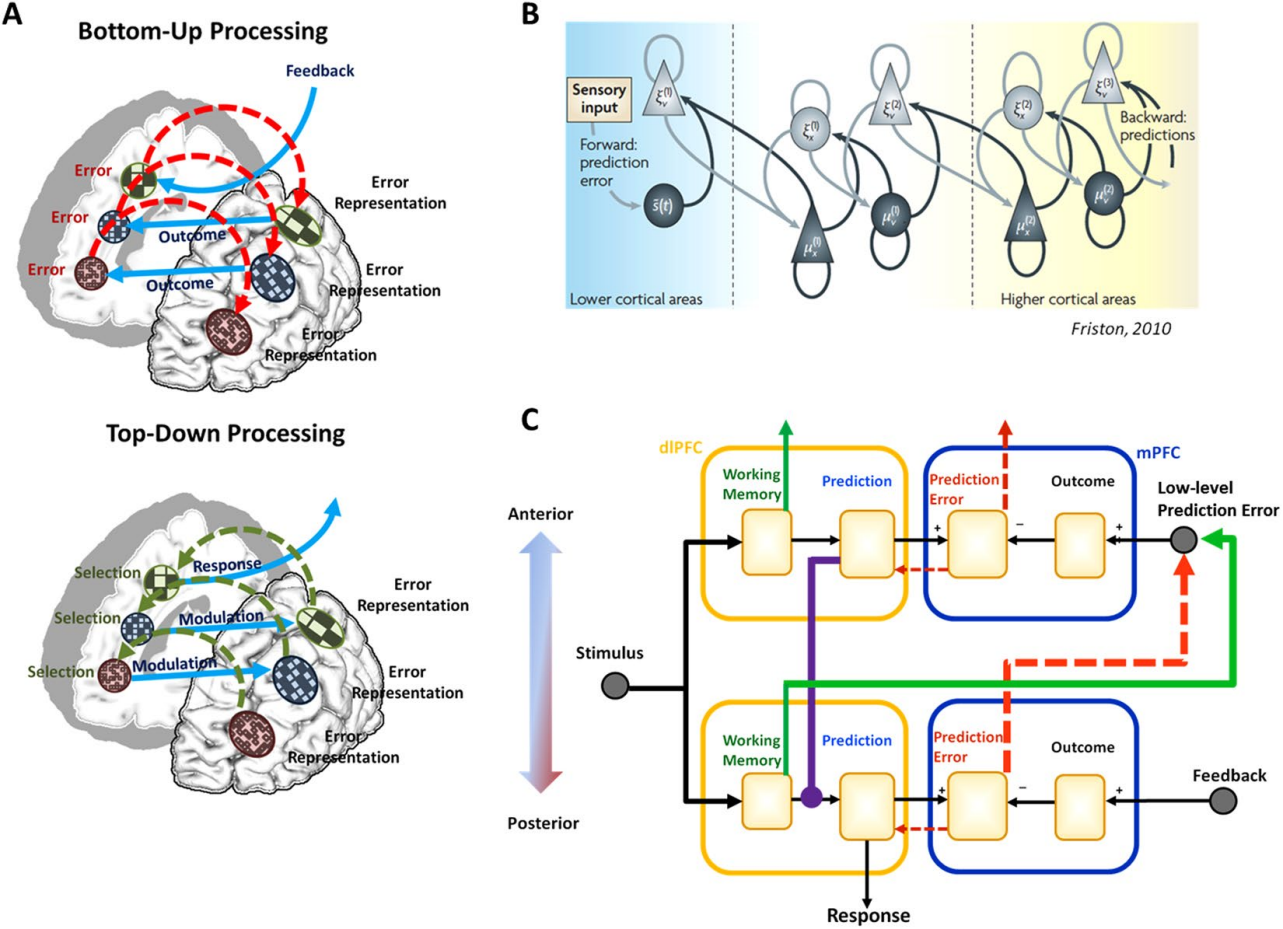
Predictive Coding in Prefrontal Cortex. (**A**) In the HER model, information is passed to hierarchical levels through bottom-up and top-down pathways. In the bottom-up paths (top), regions in mPFC compute an error signal as the discrepancy between the expected and actual output of inferior hierarchical levels. Error signals generated by mPFC train error predictions in lateral PFC which are associated with task stimuli that reliably precede them. Following training, learned representations of error predictions are elicited by task stimuli and actively maintained in dlPFC for as long as they have predictive value. In the top-down pathway (bottom), error predictions are passed from superior hierarchical levels in order to successively modulate predictions made at inferior levels. (**B**) The organization of the HER model is similar to formulations of predictive coding and free energy previously used to explain results from early sensory processing areas and hypothesized to extend into the frontal lobes. Figure reprinted with permission from Friston^14^. (**C**) A detailed circuit diagram of the HER model shows bottom-up (red and green) and top-down (violet) pathways, as well as the working memory gating mechanism that allows information to be maintained over extended durations. The connections match known neuroanatomy^41,42^.

In this report, we demonstrate a proof of principle that predictive coding computational models can account for a wide array of effects in the prefrontal cortex. In doing so, we propose solutions to several fundamental problems in neuroscience, especially the function of the frontal lobes and the nature of the representation in PFC. First, we show that the *Hierarchical Error Representation* (HER) model of mPFC and dlPFC can learn to perform a diverse array of tasks that require human subjects to represent complex relationships amongst task stimuli and to maintain information over extended periods of time. At a single hierarchical level, the HER model suggests that error signals computed in mPFC^7,25,28^ can be used to train representations of the error signal in dlPFC. *Error representations* learned by dlPFC are associated with task stimuli that reliably precede prediction error signals generated by mPFC such that, on subsequent stimulus presentations, error representations maintained in dlPFC may be deployed to reduce prediction errors in mPFC. Residual errors - those that cannot be fully predicted at a given level - act as a “proxy” outcome for higher levels of a mPFC/dlPFC hierarchy, and these proxy outcomes may in turn be the targets for further prediction and error computations. The result is a self-organizing hierarchical network that learns, maintains, and flexibly switches working memory representations as a product of learning to minimize prediction error. (Fig. 1; supplementary material/methods).

The essential principle of the HER model can be distilled to this: *A major function of prefrontal cortex is learning to predict likely prediction errors*. With this approach, we show that effects observed in PFC can be derived from the manipulation of quantities related to a common neural code of prediction error, including the activity of single units, BOLD activity during the update and maintenance of working memory, and multi-variate pattern analysis. The HER model thus reconceptualizes PFC as a region involved in computing and maintaining progressively more abstract error representations in order to govern behavior in an efficient and adaptive fashion. In the framework of predictive coding, hypothetical causes used to “explain away” prediction errors reported by lower levels emerge as each hierarchical level learns representations of residual errors reported by lower levels, and the degree to which a given hierarchical level influences the processing of a lower level is proportional to error representations learned and maintained by the model (supplementary materials/methods). Essentially, the neural code in frontal cortex is formed as neurons learn to anticipate, and thus minimize, prediction errors. We have shown how prediction errors can be used to drive cognitive control signals^29^, and a neural code in terms of prediction errors contrasts notably with competing proposals that mPFC represents value or choice difficulty^30,31^, or that lateral PFC represents working memory^32^ or categorical abstractions without necessarily specifying how those are learned^33^.

Previous computational simulations of the HER model have demonstrated its ability to learn complex cognitive tasks in a manner comparable to human performance, both in terms of behavioral markers of learning as well as the speed at which such tasks were acquired^28^. The model’s ability to perform these tasks is noteworthy considering that it is composed of a repeated motif of relatively “dumb processes” organized hierarchically: individual hierarchical levels instantiate simple RL learners that receive feedback in the form of error signals generated by lower levels, and whose predictions serve to modulate lower level predictions. Nevertheless, with respect to neuroscience, previous work has not shown whether or how predictive coding models such as the HER model might account for empirical behavioral and neuroscience results in the frontal cortex. Here we demonstrate how the HER model accounts for a range of empirical findings and is thus a plausible model of frontal cortex function. Our aim here is not to exclude other models directly – instead, we show that the HER model breaks new ground as a proof of principle that empirical findings from the frontal cortex can be plausibly modeled by predictive coding mechanisms, and specifically by the HER model. As such, the model provides not only a new perspective on frontal cortex function but also one of the broadest accounts of empirical findings in the frontal cortex to date.

In order to support the claim that the HER model provides a sufficient account for the diversity of neural signals observed in PFC at the ensemble (BOLD, EEG) and single-unit levels, we apply the model to a selection of cognitive paradigms in which PFC function has been implicated. Each selected paradigm reflects a critical aspect of PFC function: maintaining hierarchical task structure (simulation 1), the nature of distributed representations in PFC (simulation 2), response profiles of individual neurons (simulations 3 & 4), the contribution of PFC to behavior (simulation 5), and how major sub-regions of PFC interact in the course of ongoing behavior and lesion-induced deficits (simulations 6 and 7). The overarching rationale, therefore, is to demonstrate that a single unifying principle, namely that of suppression of error signals, is sufficient to account for the range of neural responses observed in PFC, as well as the varieties of functions generally attributed to the frontal lobes. Our simulations use a single parameterization of the model (see Supplementary Material) that is not explicitly tailored to each experiment in order to match qualitative patterns of neural responses and behavior, providing support for the generality of error representation and processing as the underlying factor allowing the model to capture the range of results described here. By casting our hierarchical reinforcement learning approach in the framework of predictive coding, our results provide additional support for the universality of error minimization throughout neocortex, from low-level sensory processes to high-level cognitive behaviors, and suggest a common neural currency of error and error representation throughout the brain.

## Results

Here we show how the HER model can simulate and account for a variety of published empirical findings in the dlPFC and mPFC. The results reported below are by no means exhaustive. They serve to emphasize the main point that the HER model of PFC, as an instance of predictive coding formulations, is able to autonomously learn complex tasks in a manner that reproduces patterns of behavior, neuropsychological effects, and neural activity as measured by fMRI, EEG, single unit neurophysiology observed in empirical investigation. Details of the simulations can be found in the supplementary material, along with a description of the equations defining the HER model. The supplementary material also includes further simulations that demonstrate more of the explanatory power of the HER model.

### Simulation 1: Context, Working Memory, & Control

**Figure 2:**
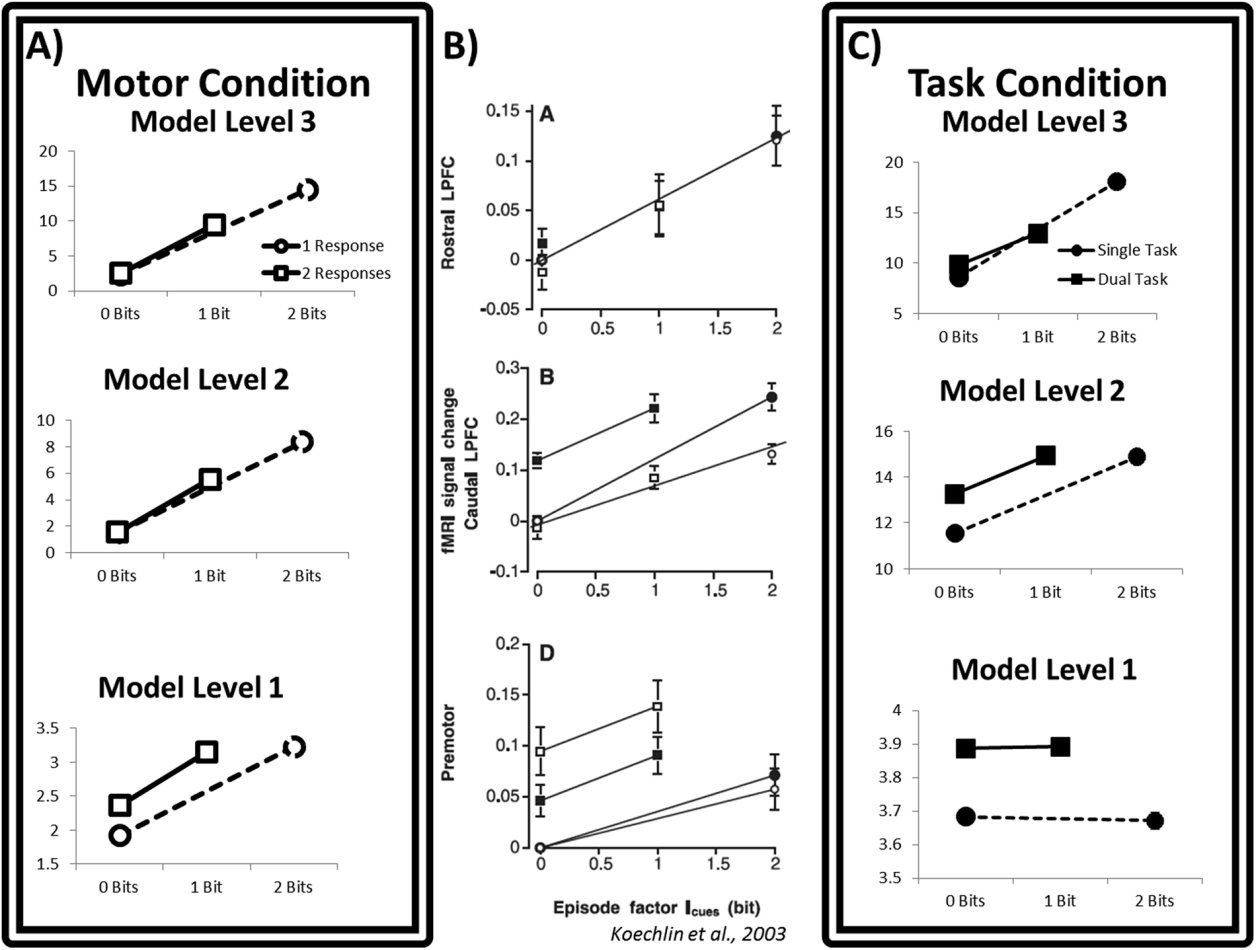
Information encoding in dlPFC. Simulated data is enclosed in double-bordered boxes throughout the manuscript. As the information content of a context cue increases, calculated in Bits (X axis), activity across hierarchically organized regions of dlPFC increases. The strength of error predictions maintained in dlPFC is proportional to information content: the more informative a cue is, the larger a reported error will be without the information supplied by that cue. (**A**) The HER model captures effects of information related both to the nature of task-relevant stimuli (x axes) as well as responses that may be required (y axes). The HER thus provides a complementary account to the Information Cascade model of PFC. (**B**) In the Task Condition of Koechlin *et al*.^24^, activity across dlPFC is observed to increase with the information content of a contextual cue. However, here activity in caudal dlPFC (panel B, middle) shows an additional increase when subjects must occasionally switch between two tasks (vowel/consonant, upper/lower case identification). (**C**) This additional increase related to task switching is accounted for as transient increases in activity in the HER model when the nature of the task changes (middle row).

The role of dlPFC in working memory and representation of task structure remains an ongoing research concern. In the past two decades, numerous fMRI studies have investigated the structure and function of dlPFC under various hierarchical task and working memory demands. In Koechlin *et al*.^24^, the authors investigated the function of dlPFC in two tasks while manipulating the amount of information conveyed by task-relevant stimuli. In their Motor Condition, activity throughout dlPFC – from areas labeled PMd (dorsal premotor cortex) to rostral dlPFC –was observed to increase monotonically as the information content of a contextual cue increased (Fig. 2B). An additional increase in activity was observed only in PMd when subjects were required to make two responses rather than a single response. In Simulation 1 (Fig. 2A,C), the HER model accounts for the general trend of increasing activity across dlPFC as the increasing strength of error prediction representations learned by the model – more information means more potential errors that must be accounted for. Summary model activity for each condition correlates with BOLD signal change observed in humans data for both the Motor condition (r = 0.70, p < 0.001) and the Task condition (r = 0.75, p < 0.001). This account complements the Information Cascade model^24^ based on information theoretic formulations; in information theory, information is the amount by which uncertainty about a random variable decreases given another variable. Error predictions learned by the HER model are used to modulate outcome predictions in order to support correct behavior - that is, their role is to reduce uncertainty regarding the likely outcomes of actions. The HER model accounts for the additional increase in activity observed in PMd through the transient update of representations (see supplementary material) at the lowest model level when successive stimuli mandate different responses, while conditions in which only a single response is required do not entail an additional update (Fig. 2A, bottom).

### Simulation 2: Learned Representation

While the HER model is able to capture a range of results related to the activity of ensembles of neurons reflected by the BOLD signal (see supplementary material), it also posits a particular representation scheme deployed in dlPFC. Namely, single units in the HER model dlPFC each code for a component of a multi-dimensional error prediction. In addition to capturing data related to the strength of activity observed in dlPFC, then, the HER model should also be able to account for data relating to the activity of individual neurons as well as techniques designed to decode neural activity such as MVPA.

**Figure 3:**
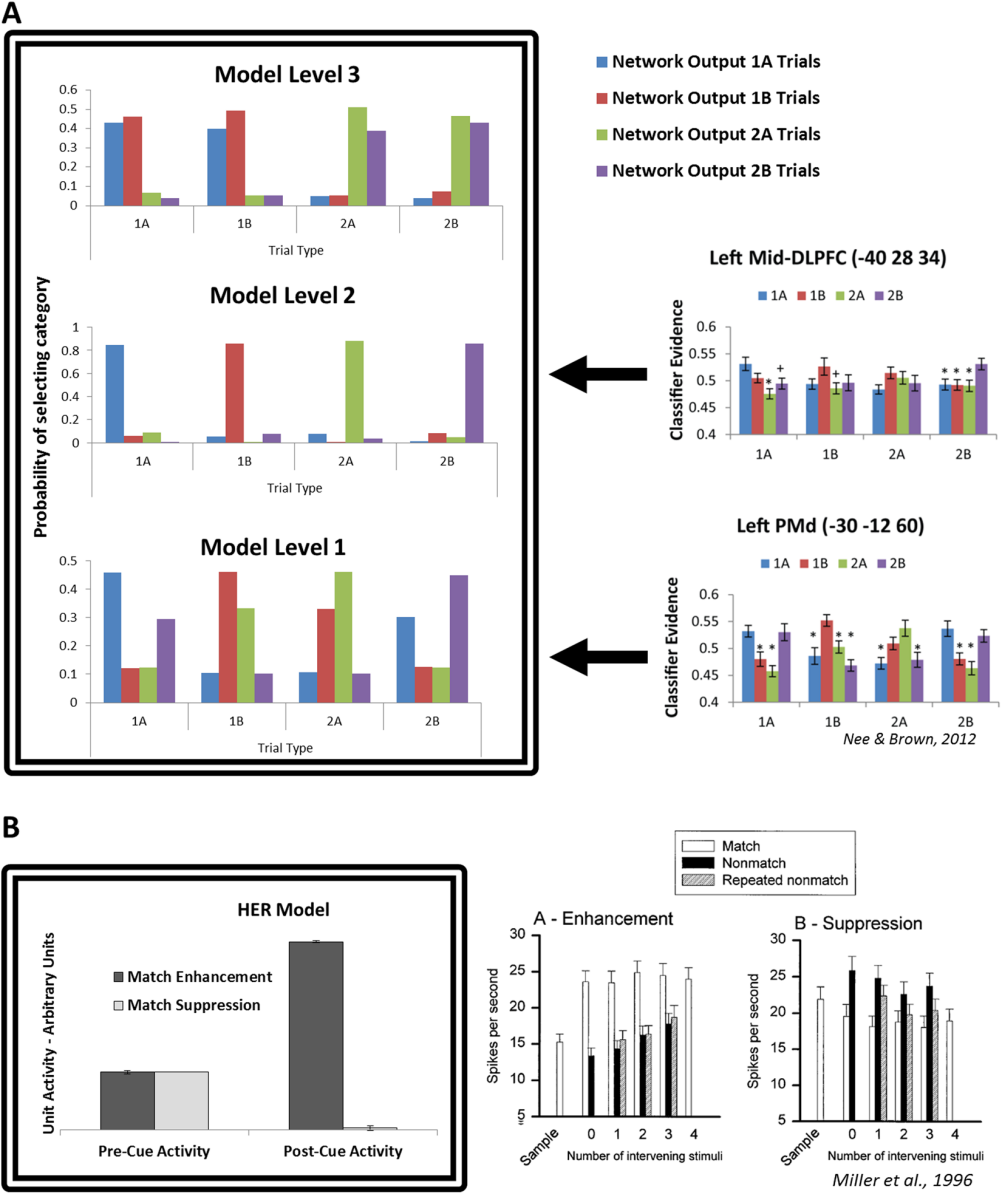
Distributed Representations in PFC. Separate units in the HER model represent components of a hierarchically-elaborated, multi-dimensional error prediction, suggesting how cognitive tasks may be represented neurally. (**A**) Left: MVPA on error prediction representations maintained by the model while performing the 1-2AX CPT are consistent with human data showing that caudal regions of lPFC code for potential target sequences regardless of higher-order context, while more rostral regions encode more abstract context variables. Right: Human MVPA results, reprinted by permission from Nee & Brown^11^. Classification results of model representations are naturally more robust than pattern analysis of fMRI data since it is possible to record the activation of units in the model with perfect fidelity, while BOLD signals are subject to noise. Nevertheless, classification accuracy for model representations was significantly correlated with classification accuracy for human data at both hierarchical level 2/mid-DLPFC (r = 0.64, p = 0.0074) and level 1/dorsal premotor cortex (r = 0.91, p < 0.001). (**B**) Units in level 1 of the HER model (left) show activity related to match suppression and enhancement while performing a delayed match-to-sample task. Prior to observing a target stimulus, activity in these units reflects the equal probability of observing a match or non-match cue. Following the presentation of the target stimulus, the activity of units predicting the occurrence of a match is enhanced, while the activity for non-match-predicting units is suppressed, similar to data recorded from monkey lPFC (right). The HER model further predicts the existence of units showing effects of mismatch enhancement and suppression. Reprinted by permission from Miller *et al*.^34^.

To investigate whether the error prediction representations learned by the HER model are consistent with those observed in human subjects, we recorded activity from the model as it performed the 1–2AX continuous performance task (Simulation 2, Fig. 3A). We subsequently classified active representations in the model during periods of the task in which the model had been shown high- and low-level context variables (see Online Methods), but prior to a potential target cue being displayed. This approach is similar to the multi-voxel pattern analyses reported by Nee & Brown^11^. Classification of the model representations is consistent with that observed in human subjects (Fig. 3A): at the lowest hierarchical level, sequences that may culminate in a target response (1 A/2B) and those that will certainly not culminate in a target response (1B/2 A) are represented in a distinct fashion (Fig. 3A, Bottom). However, the representations also partially overlap such that 1 A sequences are partially categorized as 2B sequences, while 1B sequences are partially categorized as 2 A sequences. At level 2 of the HER model, classification of each sequence is more decisive, with each unique sequence (1 A/1B/2 A/2B) being unambiguously decoded (Fig. 3A, Middle). This result is similar to human data, in which a region in mid-dlPFC shows a trend toward increased evidence for unique sequence coding. Finally, at the third hierarchical level (Fig. 3A, Top), sequences beginning with 1 or 2 are each collapsed (i.e., equal evidence for 1 A and 1B), reflecting the role of rostral dlPFC in coding high level context variables. The HER model explains the confusion of one target sequence with another (1 A/2B) and one non-target sequence with another (1B/2 A) at the lowest hierarchical level as a consequence of the increased activation of a predicted response common to both types of sequences – a target response in the former condition, and a non-target response in the latter condition.

### Simulation 3: Single-Unit Neurophysiology

The representation scheme proposed by the HER model suggests that individual neurons in lPFC should code for components of a distributed error representation, with single units signaling the identity and likelihood of observing a particular error. The model further suggests that these signals should evolve through the course of a trial as the likelihood of observing specific types of errors increases or decreases. We recorded activity in the model as it performed a delayed match-to-sample (DMTS) task (Simulation 3). Consistent with observed unit types recorded in macaque monkeys^34^, units in the HER model were identified with increased activity following the occurrence of a target probe that matched the sample (match enhancement; Fig. 3B), while distinct units were identified whose activity decreased following a matching target (match suppression; Fig. 3B). The HER model accounts for these two types of neurons as the modulation of predictions regarding possible responses following the presentation of a target cue. When a matching target is presented, the activity of units predicting a “match” response increases (enhancement) while the activity of units predicting a “non-match” response decreases (suppression). The HER model further suggests *a priori* that additional types of neurons should be observed in lPFC, namely *mismatch* enhancement and suppression neurons – neurons whose activity reflects the increased and decreased likelihood of making a non-match and match response, respectively.

### Simulation 4: Mixed Selectivity

**Figure 4:**
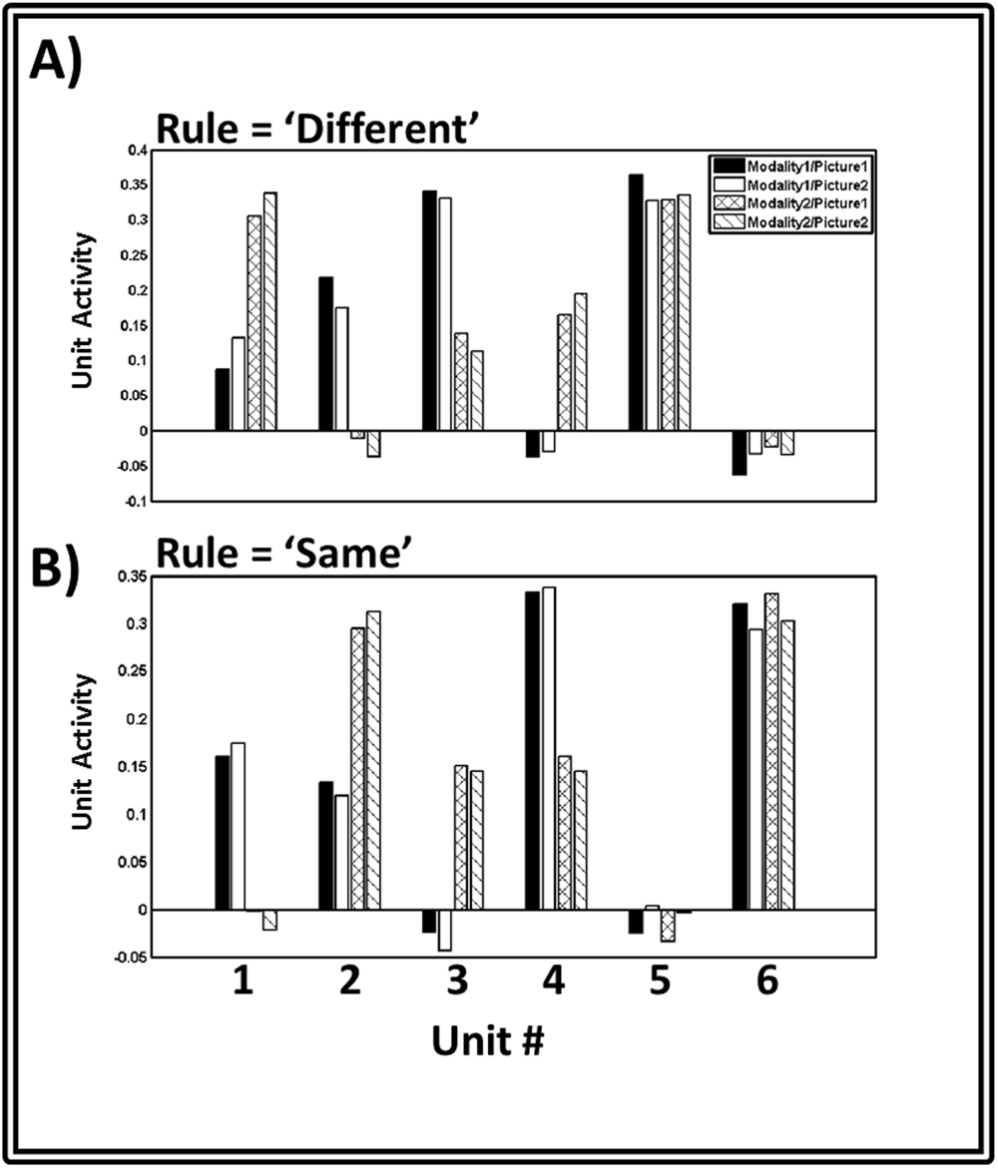
Mixed Selectivity in the HER model. Units from level 2 of the HER model show complex patterns in response to stimuli. The responses of units 1–4 show interactions between a cued rule and a sample stimulus presented during a DMTS task, with some units preferentially responding to, e.g., combinations of ‘Same’ rules and ‘Picture 1’. Additional units in the model (5 & 6) respond solely to a preferred rule: ‘Same’ (Unit 6, panel B) or ‘Different’ (Unit 5, panel A). The combination of rule-specific and ruleXcue interaction units replicates similar findings in primate LPFC^36^.

A further test of the error representation scheme postulated by the HER model is to examine whether the error representations learned by the model can explain the diversity of neuron types commonly observed in single-unit neurophysiological studies. Single neurons in PFC routinely exhibit mixed selectivity^35^, responding in a heterogeneous fashion to combinations of task-relevant stimuli. To investigate whether units in the HER model exhibit mixed selectivity, we simulated the model on a variation of the DMTS task^36^ in which the sample and target probes were preceded by a rule cue indicating whether the model should make a target response to MATCHING sample/target combinations (as in the usual DMTS), or whether the model should make a target response to NON-MATCHING sample/target combinations. Model activity recorded from level 2 of the HER hierarchy reveals a cluster of 6 units whose activity was reliably associated with task performance (Fig. 4). Two of these units responded exclusively to the rule cue - one unit was active following MATCHING cues, and silent for NON-MATCHING cues, while the other showed the opposite pattern. The remaining units exhibited complex patterns of activity across rule, modality, and picture identity conditions, consistent with neuron types observed in primate PFC.

### Simulation 5: The neural bases of behavior in prefrontal cortex

**Figure 5:**
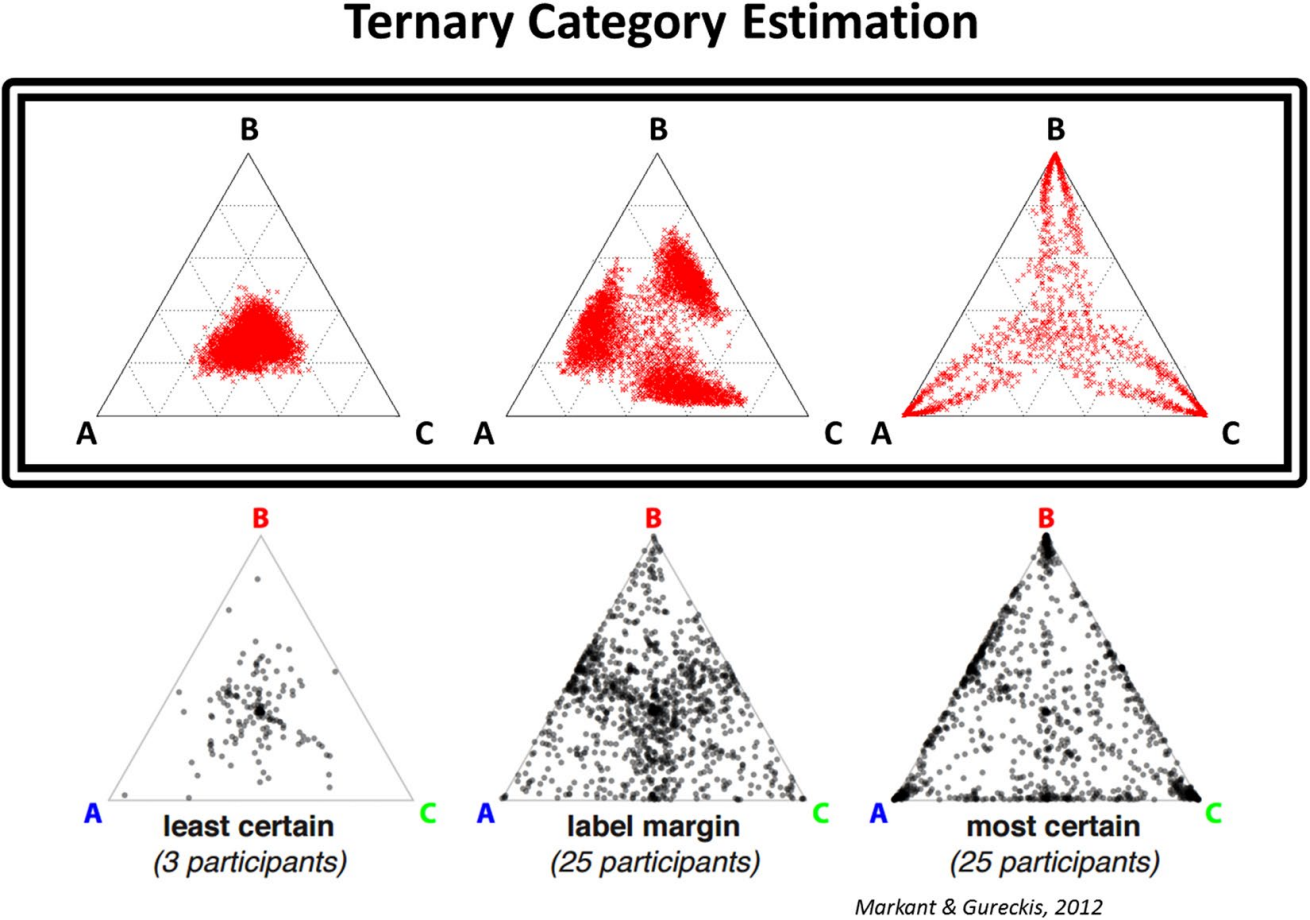
Connecting representations to behavior. Behavior of the HER model (top) with learning selectively enabled at zero (left), one (center), and all (right) hierarchical levels. Each point represents a single trial. The model’s estimate of the probabilities of three possible categories matches the behavior of three groups of human subjects with varying information sampling strategies (bottom) during a ternary probability estimation task. The HER model thus provides an account of how task representation at the level of single units contributes to behavior. Reprinted by permission from^37^.

In addition to reproducing effects from human fMRI data and single-unit neurophysiology studies in monkey regarding the nature of representations in PFC, the HER model also suggests how these representations may influence patterns of behavior. In order to investigate the influence of hierarchically-organized representations on the timecourse of learned behaviors, we simulated the model (Simulation 5, Fig. 5) on a ternary probability estimation task^37^ in which subjects were asked to estimate the probability that a compound stimulus, varying along two feature dimensions, belonged to each of three categories. Our simulations differ from the original task in that, in the human experiment, subjects were allowed to choose samples from a two-dimensional problem space, whereas in our simulations, the model was shown randomly selected samples. Nonetheless, the target behavior of both the experiment and our simulations was the same, namely probability judgments of categories. Human subjects were found to adopt three different strategies in their probability judgments corresponding to their sampling behavior (Fig. 5, bottom row): one group (Least Certain, LC, left) consistently assigned near-equal probabilities for each category, a second group (Label Margin, LM, center) assigned a low probability to one category and approximately equal probabilities to the other two, while the final group (Most Certain, MC, right) assigned a high probability to one category and low probabilities to the others. Similar patterns of behavior were observed in the HER model during simulated experiments in which the learning rate was manipulated as follows (Fig. 5, top row). For simulations in which all learning was disabled, the model’s probability estimates corresponded to the LC group. When learning was enabled only for the lowest hierarchical level, the model’s behavior corresponds to the LM group, reflecting learned representations that allow the model to rule out one of the three categories but lacking the higher order information required to distinguish between the remaining two. Finally, when learning is enabled for all levels, the model rapidly learns the entire task, corresponding to the behavior of the MC group. In the HER model, these behaviors are intimately linked to learned error predictions: the model decomposes a task by selecting, at each hierarchical level, the stimulus feature that best reduces response uncertainty. In this latter case, model behavior progresses rapidly through the behaviors associated with disabling learning at successive stages: initially the model’s behavior corresponds to the LC group, followed by LM, before converging on a solution to the ternary estimation problem, suggesting how realistic learning may require the acquisition of low-level associations prior to the development of higher-level representations. The HER model thus provides an account of how neural representations acquired during learning might contribute to patterns of behavior - the inability to form higher-order representations not only influences probability judgments, but may additionally inform self-directed sampling of information.

### Simulation 6 & 7: Interaction of mPFC and dlPFC

**Figure 6:**
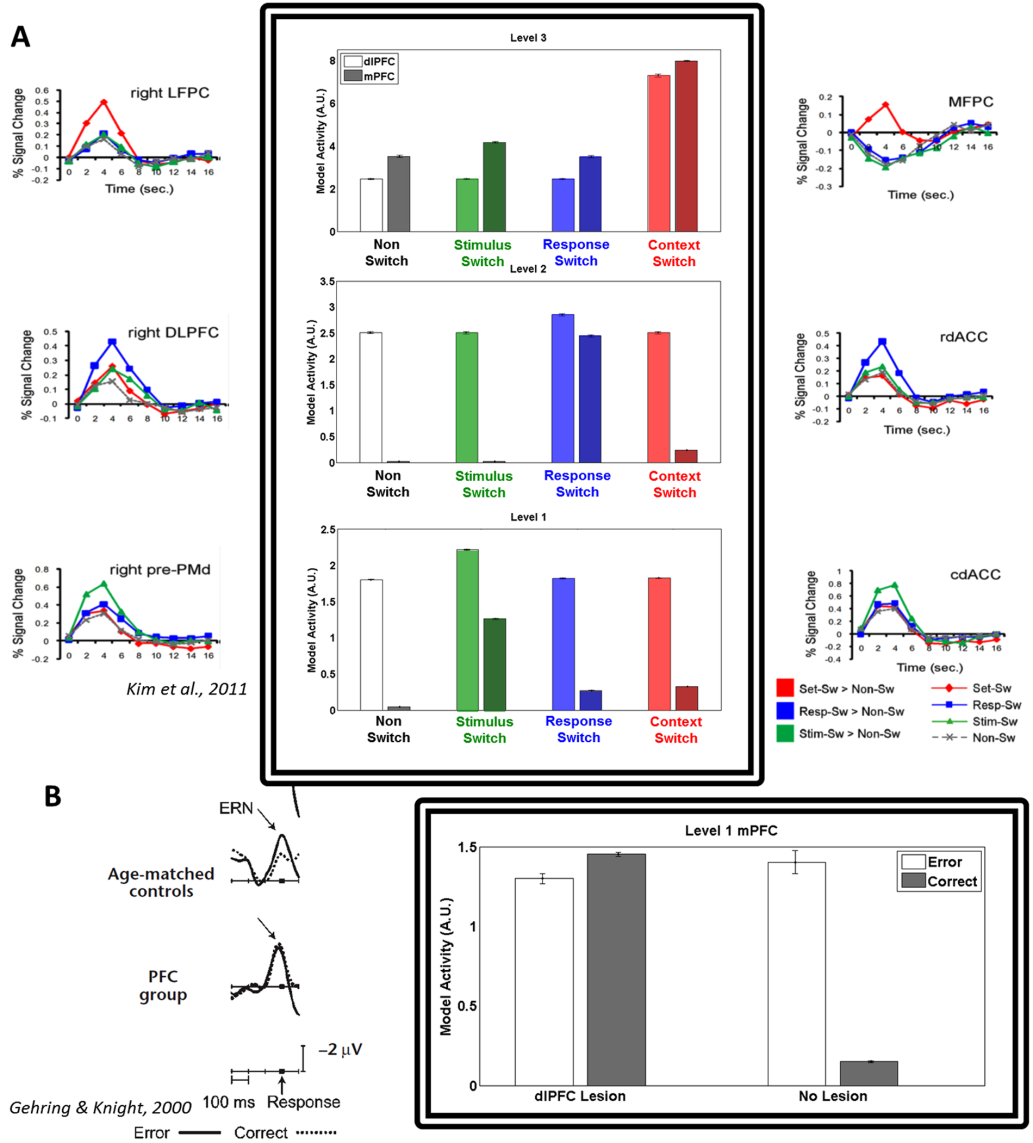
Interactions of mPFC and dlPFC, Simulations 6 and 7. The HER model suggests how mPFC and dlPFC may cooperate to minimize prediction error through passing error and error prediction information through hierarchical levels. (**A**) **Simulation 6**. Increased activity in parallel hierarchical regions in the HER model, associated with mPFC and dlPFC, is associated with errors (mPFC) and updates of error predictions (dlPFC) at different levels of abstraction, from concrete (level 1, stimulus switch) to abstract (level 2, response switch; level 3, context switch). (**B**) **Simulation 7**. Modulation of mPFC by error predictions maintained in dlPFC is critical for contextualizing predictions regarding the likely outcome of actions. In a delayed-match-to-sample task, the HER model correctly captures the elimination of the ERN following correct trials due to the maintenance of information regarding the sample cue. However, when the model is lesioned such that information normally maintained in dlPFC is no longer available to mPFC, the model produces an ERN to correct and error trials alike.

The HER model, being an extension of the predicted response-outcome (PRO) model of ACC/mPFC, already captures a wide array of effects observed within ACC^7,25^. The HER model extends beyond the PRO model in two critical ways: firstly, it specifies how mPFC and dlPFC may interact in order to support sophisticated behaviors, and secondly, it suggests a parallel hierarchical organization of mPFC in which successive hierarchical regions report increasingly abstract error signals. Such an organization of mPFC has been proposed previously^38,39^, and, indeed, evidence has been found that supports a role for mPFC in processing hierarchical errors^27^. The HER model is able to capture the pattern of activity observed by Kim *et al*.^26^ (Simulation 6) for distinct regions of both mPFC and dlPFC (Fig. 6A, middle column). The HER model interprets activity in hierarchically-organized regions of mPFC as the discrepancy between increasingly abstract predicted and observed outcomes, consistent with the role of mPFC in error computation proposed by the PRO model^7,25^, and complementary to the interpretation of Kim *et al*. However, while their notion of higher-order error signals is specified qualitatively, successively more abstract errors in the HER model are a product of quantitative predictions at lower levels that are insufficient to explain a subject’s observations, in line with the predictive coding framework that informs the structure of the HER model.

Additional evidence regarding the interaction of mPFC and dlPFC comes from studies of patients with dlPFC lesions^40^. In a delayed match to sample task, an Error Related Negativity (ERN) is observed in subjects with lesions to dlPFC for both correct and incorrect trials (Fig. 6B, left column). The HER model (Simulation 7, Fig. 6B) explains this as the inability to maintain relevant information across a delay period in order to modulate predictions regarding likely outcomes (Fig. 6B, right column). Without this additional contextual information available in the model, both correct and incorrect outcomes are surprising, resulting in increased mPFC activity in a lesioned version of the HER model on both types of trials.

## Discussion

In this paper, we have deployed a new computational neural model, consistent with known anatomy^41,42^, to simulate a range of effects observed in studies of mPFC and dlPFC. Simulations demonstrate that the HER model captures various dlPFC effects, as well as how dlPFC and mPFC interact to support the acquisition and execution of sophisticated cognitive tasks. Because the HER model extends our previous PRO model of ACC/mPFC^7^, it can also comprehensively account for mPFC activity in simple cognitive control experiments as previously reported^7,25^. These results, taken as a whole, make the HER model among the most comprehensive models of PFC to date and provide a process model proof of principle that predictive coding formulations, coupled with representations based on the computation and manipulation of quantities derived from error, can account for a large corpus of PFC empirical findings.

The HER model provides a complementary perspective on existing models. Donoso *et al*.^43^ cast the PFC as searching for, evaluating, selecting, and discarding task strategies to maximize reward. In the HER model, task strategies are represented automatically as hierarchical self-organized abstract representations of task context, which serve as a working memory basis for guiding behavior. Strategies are discarded from working memory when they no longer provide useful predictive information about subsequent events, or when contingencies change such that predictive information in working memory is repurposed by retraining its connections to modulate lower level predictions differently. The HER model can switch strategies flexibly as task cues change, and it can learn new responses when environmental contingencies changes. As with other neural models that include PFC^44^, as well as models of hierarchical behavior^4,45^, the HER model captures key aspects of neural anatomy, neurophysiology, and behavior during performance of cognitive tasks. The HER model further addresses the question of how these tasks might be learned in the first place, as well as how the components of a task are represented as expected prediction errors. The HER model thus fills a critical void left by models concerned with how coherent behaviors are organized based on pre-existing representations without specifying the nature of those representations or how those representations were acquired^4,44,45^.

The HER model also addresses questions of how representations are gated into task-relevant prefrontal working memory. While other models posit reinforcement learning of what to store and when to allow stored elements to be output to other regions in order to maximize value^46,47^, the HER model rather learns what to store in order to minimize prediction errors. In this respect it performs a similar function as LSTM^48^, although the mechanisms of the HER model are entirely different.

More generally, the HER model demonstrates how the predictive coding framework may be extended into prefrontal cortex in order to account for sophisticated cognitive behaviors. The HER model inherits many of its formalisms from hierarchical RL - each hierarchical level of the HER model is a relatively straightforward RL learner based on previous models of mPFC^7,25^, and augmented with a WM component able to maintain representations over periods of time^49^. Learning at each level of the model proceeds from the need to suppress the prediction errors signaled by lower levels, as in predictive coding accounts of perceptual inference^50^. It is notable that the model is not only able to replicate effects observed throughout PFC during the performance of complex tasks, but it also learns these tasks autonomously in a manner comparable to human performance^28^, despite its simple motif structure. By showing how models of tasks might be learned incrementally through the principle of suppressing prediction errors, the HER model provides a complementary account to approaches such as active inference^51^ which have been leveraged to explain neural activity as minimizing surprise by inferring states of previously learned models^52^. While not strictly performing active inference, the HER model (once trained) does in a sense infer latent states by storing corresponding external cues in working memory. These working memory activations constitutes a *de facto* representation of inferred states and thus provides a context-dependent pattern of activity that minimizes prediction error. More broadly, the HER model extends predictive coding formulations in two key ways, both as an account of the function of the frontal lobes, as well as a plausible mechanism for learning models of the world, and in doing so, provides additional evidence in support of error minimization as a fundamental principle of brain function.

## Electronic supplementary material

Methods, Supplementary Results & Discussion
