# Supplementary material for: Frontal cortex function as derived from hierarchical predictive coding
Source: Sci Rep. 2018 Mar 1;8:3843. doi: 10.1038/s41598-018-21407-9 (PMC5832795; doi:10.1038/s41598-018-21407-9)
Supplement: Supplementary file 1 — Methods, Supplementary Results & Discussion [file 41598_2018_21407_MOESM1_ESM.docx]

Frontal cortex function as derived from hierarchical predictive coding: Methods, Supplementary Results & Discussion

| William H. Alexander^1^  Ghent University | Joshua W. Brown^2^  Indiana University, Bloomington |
| --- | --- |

Running Title: Frontal Cortex Function

For Submission to: Scientific Reports

January 9, 2018

Keywords: computational models, anterior cingulate, dorsolateral prefrontal cortex, working memory, cognitive control.

^1^Ghent University

^2^Indiana University, Bloomington

**Address correspondence to:**

William Alexander (william.alexander@ugent.be)

**Methods**

*The Hierarchical Error Representation Model*

A detailed description of the HER model is provided in our previous publication^1^, and selected simulation code for the present paper is available at https://github.com/modelbrains/HER_model. Here we provide a summary of the components of the HER model and how they interact (Fig. S1). The HER model is composed of multiple levels, each instantiating a relatively simple RL learner based on the Predicted Response-Outcome (PRO) model of mPFC^2^, and endowed with a working memory gating mechanism that governs whether a stimulus is stored in WM or not. Levels interact with one another through top-down and bottom-up pathways. As in predictive coding, the objective of the HER model is to minimize prediction error. Locally, each hierarchical layer attempts only to minimize its own prediction errors through update of associative weights linking WM representations to predictions, as well as weights determining when the contents of WM are updated with new information. Global error minimization is achieved by the top-down and bottom-up interactions of hierarchical layers - errors that cannot be explained at one hierarchical level are passed up the hierarchy in order to train predictions at superior hierarchical levels; these predictions are then used to modulate predictions at inferior levels. The minimization of global prediction error is thus a product of local processes that attempt only minimize their own error signals.

*
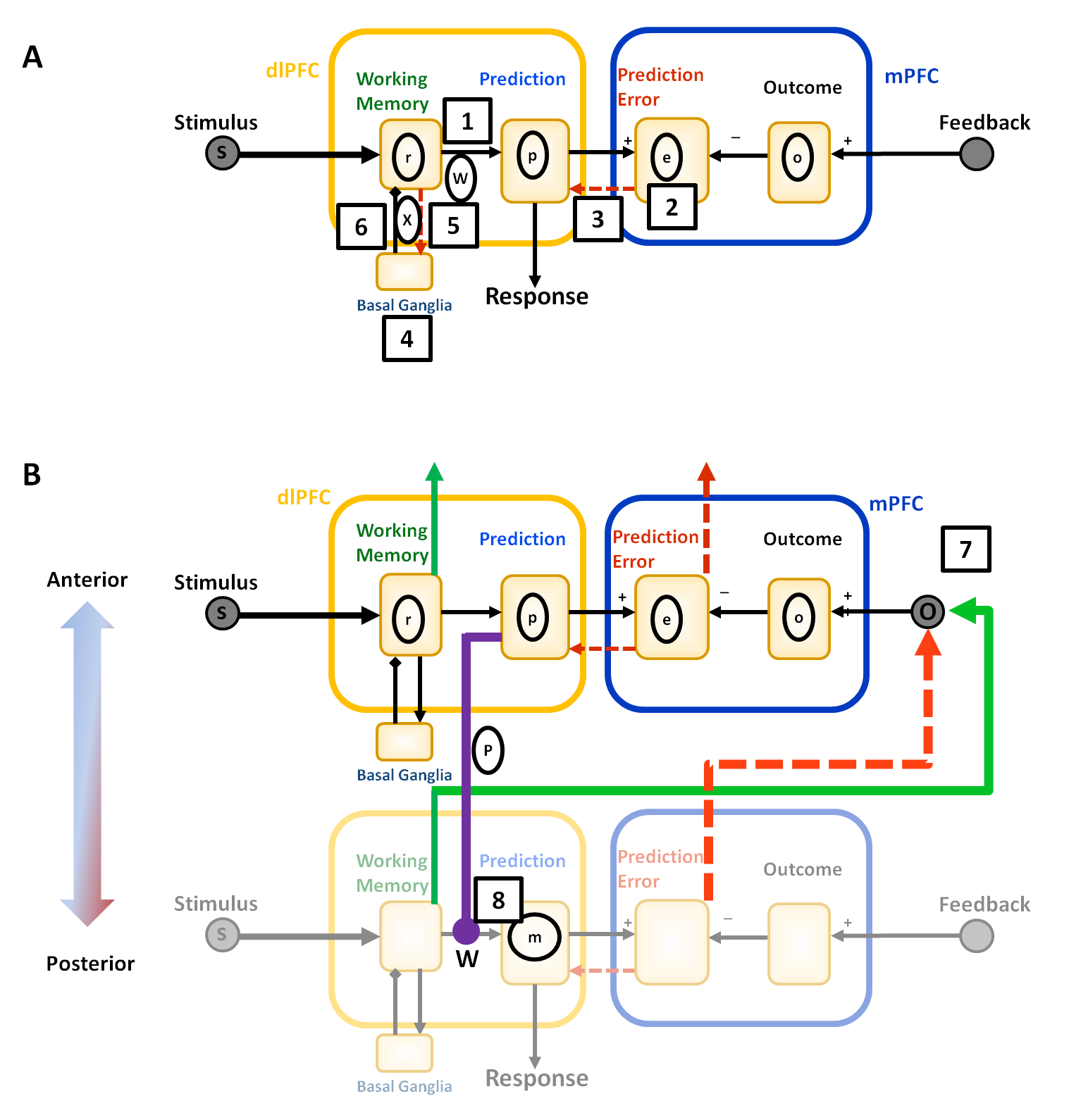
*

**Figure S1. Annotated model schematic.** Relevant model variables are within ovals, and equations are in boxes. **A)** **Within-level model function.** Each hierarchical level of the HER model functions as the PRO model, with the addition of a working memory mechanism. **B) Interactions between levels.** Error signals generated by each hierarchical level are passed to superior hierarchical levels as a training signal. Predictions learned at superior hierarchical levels modulate predictions generated at inferior levels.

*Reinforcement Learning*

The output of each level is determined by the item currently stored in WM at each level and the strength of weights associated with that item:

$\mathbf{p}\mathbf{=}\mathbf{W}^{\mathbf{T}}\mathbf{r}$ (1)

where **p** is a vector of predicted outcomes, **r** is the item currently stored in WM, and **W** is a weight matrix associating **r** and **p**. Errors at each level are computed as the difference between observed and predicted outcomes:

$\mathbf{e}\mathbf{=}\boldsymbol{a\circ}\left( \mathbf{o}\mathbf{-}\mathbf{p} \right)$ (2)

where **o** is the observed outcome and **a** is a filter (of the same dimensionality as **o** and **p**; the symbol $\boldsymbol{\circ}$ denotes element-wise multiplication) set to 0 for unselected actions and 1 everywhere else, effectively preventing learning about unselected actions in the model. Weights are updated according to:

$\mathbf{W}_{t+1}\boldsymbol{=}\mathbf{W}_{t}\mathbf{+}\alpha(\mathbf{e}_{t}{\mathbf{r}_{t}}^{\mathbf{T}}\mathbf{)}$ (3)

where $\alpha$ is a learning rate parameter and *t* indicates the current model iteration.

*Working Memory Gating*

The WM gating mechanism, inspired by models of basal ganglia^3^, determines whether a currently presented stimulus will be stored in WM at each level. This determination is made based on the learned, relative value of encoding a new stimulus in WM vs. maintaining the current contents of WM:

$\mathbf{v}\mathbf{=}\mathbf{X}^{\mathbf{T}}\mathbf{s}$ (4)

Here **s** indicates a vector of external stimulus features (distinct from internal representations **r**), **X** is a weight matrix associating stimulus features with WM representations, and **v** is the value of storing a particular feature in **s** as a representation in working memory **r**. Weights **X** are trained through backpropagation of the error term calculated in eq. 2:

$\mathbf{X}_{t+1}\boldsymbol{=}\mathbf{X}_{t}\mathbf{+}(\mathbf{e}_{t}^{\mathbf{T}}\mathbf{W}_{t}\boldsymbol{\cdot}\mathbf{r}_{t})\mathbf{d}_{t}^{\mathbf{T}}$ (5)

Rather than directly associating WM representations **r** with currently present external stimuli (**s)**, the update rule associates a temporally-extended eligibility trace of stimuli that have been observed recently, **d.** On each model iteration, **d** is multiplied by a scalar decay parameter, λ (see table 1), and elements of **d** that correspond to currently present stimuli are set to 1.

While error backpropagation of error is biologically implausible, our intent was not to develop a neurally faithful model of basal ganglia, and so backpropagation was selected for computational convenience. Nevertheless, in our previous work, we demonstrate how a more realistic model of WM gating using scalar reinforcement signals may be implemented in a manner consistent with previous proposals ^3–5^, so that the HER model functions as well while maintaining biological plausibility.

The value of storing stimulus features in WM is passed through a softmax function in order to determine whether a stimulus will be stored in WM or the current contents of WM will be maintained.

$probability of storing s_{i} = \frac{(\exp^{\beta v_{i}})}{{(exp}^{\beta v_{i}})+(\exp^{\beta v_{j}})}$ (6)

$\beta$ is a gain parameter governing the probability of selecting the highest value in **v**. In the framework of predictive coding and free-energy^6^, the softmax gain parameter is frequently referred to as a 'precision' reflecting the degree of confidence in the expectation of a policy^7^. In our simulations, only a single stimulus was maintained in WM at each level on any model iteration, and only one stimulus was presented. The softmax equation therefore operates only over the elements in **v** that correspond to the currently active WM representation (v_j_) and the current stimulus feature (v_i_).

*Bottom-Up and Top-Down Pathways*

The RL algorithm and WM gating mechanisms operate at each level of the HER model. Layers interact with one another through bottom-up and top-down pathways based on Predictive Coding formulations. In the bottom-up pathway, errors reported by a given hierarchical level (eq. 2) are passed to a superior hierarchical level. In the HER model, the error reported by a level, conjoined with active WM representations at that level, acts as the outcome for the next higher level:

$\mathbf{O}=\mathbf{r}\mathbf{e}^{\mathbf{T}}$ (7)

Here **O** is the matrix computed from the outer product of the error and WM representation vectors. For computational convenience, this is reshaped into a vector **o**. Error computation and learning is identical at each level (eqs 2 & 3), with the exception that the outcome term at each level above the 1st is derived from eq. 7.

The purpose of training higher-order levels in the hierarchy using the outcome term in eq. 7 is to derive predictions regarding the likely errors that can be expected at lower-order levels. Predictions at each level are calculated as in eq. 1; however, at higher-order levels, these predictions reflect expected errors reported by lower levels. Since knowledge of likely errors can be useful in avoiding those errors, the predictions generated at each level can be used to modulate the predictions generated by inferior levels. For levels above the 1st, the prediction **p** is reshaped into a matrix **P** of the same dimensionality as the weight matrix **W** of the immediately inferior level. **P** and **W** are then added to one another, resulting in a modulated weight matrix used to compute a prediction of likely outcomes that incorporates higher-order information:

$\mathbf{m}\boldsymbol{=}\left( \mathbf{W}\mathbf{+}\mathbf{P} \right)^{\mathbf{T}}\mathbf{r}$ (8)

where **m** is the modulated prediction. The higher-order information added to **W** by **P** amounts to conditioning the associations contained in **W** based on WM representations maintained at the superior level.

*Responses*

At the base level, model activity is translated into response probabilities. As in the PRO model^2^, the HER model learns predictions of response-outcome associations. Individual responses may be associated with either correct or error feedback. In order to generate a response, the learned likelihood of receiving correct feedback is compared to the learned likelihood of receiving error feedback for each candidate response:

$u_{response}= m_{Response/Correct} - m_{Response/Error}$ (9)

which is then passed through a softmax function to determine the probability of the model making each response:

$Prob.(u_{i})=\frac{{exp}^{\gamma u_{i}}}{\sum{exp}^{\gamma\mathbf{u}}}$ (10)

where $\gamma$ is a gain parameter.

In the HER model, stimuli **s** and outcomes **o** are both the product of sensory observations. In our simulations, we generally treat stimuli as observations that require a subsequent response, while outcomes are observations that follow a response. In previous work^8^, we note that this distinction is somewhat arbitrary - effects observed in mPFC can be derived by treating stimuli and outcomes as similar sensory entities. However, in order to simulate the array of results presented here, we adopt the ubiquitous perspective of stimuli preceding, and being causally related to, outcomes.

*Sources of Activity in Prefrontal Cortex*

A key concern in relating computational neural models to empirical data, especially with regard to indirect measures of neural activity such as EEG and BOLD signals, is in selecting an appropriate measure of model activity. In the PRO model^2^, ACC/mPFC activity was interpreted as negative surprise – the ongoing difference of predicted outcomes minus actual observations. In the HER model, the role of mPFC is identical to its function in the PRO model, and thus the measure of model activity for mPFC remains the same:

$ACC=\sum_{i} \left| {Predicted Outcome}_{i} -{Observed Outcome}_{i} \right|$ (11)

In comparison, dlPFC is thought to have multiple underlying mechanisms that contribute to its temporal activity profile. First, and central to its role in working memory, sustained dlPFC activity is observed during maintenance periods of a task when one or more items must be remembered in order to inform future behavioral responses. While recent evidence suggests that sustained activity is not a requirement for storing information^9,10^, there is nevertheless a reliable correlation between WM and elevated BOLD activity in dlPFC during maintenance periods^9^. Second, the process of encoding an item in WM corresponds to a transient increase in BOLD activity following the presentation of an item to be maintained in WM. Finally, the level of sustained activity observed in dlPFC is additionally modulated by higher order information.

The role of dlPFC in the HER model is to learn to represent task stimuli that reliably precede prediction error signals generated by ACC. That is, dlPFC learns the expected error given a current WM representation **r**:

${Error Prediction}_{i}=E\left[ \mathrm{ACC}_{i}|\mathbf{r} \right]$ (12)

Using Error Prediction as the unit of currency, then, we model the three sources of dlPFC activity described above as follows. First, sustained dlPFC activity related to WM maintenance is calculated as the absolute value of active error predictions:

$\mathrm{DLPFC}^{\mathrm{Maintenance}}=\sum_{i} \left| {Error Prediction}_{i} \right|$ (13)

Second, transient activity related to updating the contents of WM is modeled as the absolute difference on successive model iterations, t-1 and t, of active error predictions:

$$\mathrm{DLPFC}_{t}^{\mathrm{Update}}= \sum_{i} \left| {Error Prediction}_{i,t}{-Error Prediction}_{i,t-1} \right|$$

(14)

Finally, the influence of top-down information on sustained activity is modeled as the difference between the Error Prediction for a given level, and the Error Prediction at that level if there were no top-down modulation of error predictions:

$\mathrm{DLPFC}^{\mathrm{Modulation}}=\sum_{i} \left| {Modulated Error Prediction}_{i}-{Error Prediction}_{i} \right|$ (15)

While evidence has been found that dlPFC activity is sensitive to these three factors, it is likely that they contribute to activity in differing measures. However, in our simulations, we adopt a naive assumption regarding their relative contributions: we assume that the contribution of each source is simply the sum of the quantities as calculated in eqs 13-15:

$\mathrm{DLPFC}=\mathrm{DLPFC}^{\mathrm{Maintenance}}+\mathrm{DLPFC}^{\mathrm{Update}}{+DLPFC}^{\mathrm{Modulation}}$ (16)

Our rationale for this naive assumption is that, by using only the unmodified values calculated by the model, it provides a stronger demonstration of the HER model's broad ability to capture patterns of activity observed in dlPFC across a range of empirical studies, i.e., the results we report do not depend on a specific weighting of these sources of activity, nor do they depend on a parameter set specifically tailored to each experiment (cf. table 1).

Using eq. 16 as our measure of dlPFC activity, we show that the HER model is able to reproduce patterns of activity observed in hierarchically organized regions of dlPFC for tasks involving significant WM demand. Moreover, we show that the proposed role of dlPFC in learning and maintaining representations of error is sufficient to reproduce data from single-unit and MVPA studies investigating the nature of representations in dlPFC. Finally, using eq. 11 as our measure of mPFC activity, the HER model is able to capture the joint pattern of activity observed in mPFC and dlPFC data.

**Simulations**

In order to ensure that the effects reported were not due to a specific parameterization tailored to each task, all simulations were conducted using a common parameter set (table 1) unless otherwise noted below. In previous work^1^ we examined the influence of alternative parameterizations on the development of the HER model. Parameters for the current simulations were chosen based on this prior exploration in order to ensure that the model was able to learn each task, but no additional parameter adjustments were made for individual experiments (barring the learning rate adjustments noted in simulation 5). All simulations were conducted using experimental paradigms previously reported in the literature, and no new empirical data is reported in this manuscript.

*Simulation 1 : Koechlin et al (2003).*

We simulated the HER model on the behavioral task described in Koechlin (2003)^11^. For the sake of brevity, we refer the reader to the supplementary online material associated with that paper for a detailed description. Briefly, subjects participated in two experiments, a "motor" experiment and a "task" experiment. In each experiment, the subjects experienced blocks of 12 sequentially presented stimuli whose visual appearance(color) indicated which response they were to make (in the case of the motor experiment) or which task (vowel/consonant or upper/lower case discrimination) they were to perform. Each block was preceded by a context cue which indicated the mapping between stimulus color and the appropriate response or task. We simulated the motor and task conditions separately. For the "motor" experiment, inputs to the model were the 4 context cues associated with each condition, and 6 colors that were observed by the subjects during the experiment. 2 responses were possible (left or right), and feedback to the model indicated either correct or incorrect performance. For the "task" experiment, model inputs were the 4 context cues associated with each condition, 6 colors observed during the experiment, and 4 cues indicating whether the stimulus was a vowel or consonant, or upper or lower case. 4 responses could be generated by the model, indicating upper or lower case or vowel or consonant responses. Feedback to the model indicated correct or incorrect performance. The model performed each condition for 4500 blocks (1 block = 12 trials).

*Simulation 2: 1-2AX CPT*

The 1-2AX CPT^3,12^ is a hierarchically organized task in which a subject's response to a target cue ('X' or 'Y') is governed by both the cue that immediately preceded it ('A' or 'B'), as well as a "context" cue ('1' or '2') that indicates which target sequence ('AX' or 'BY') is valid at any given time. Sequences of stimuli may be thought of as being organized in 'inner' and 'outer' loops, where inner loops are composed of 2-stimulus sequences with 'A' or 'B' followed by 'X' or 'Y', and outer loops are the sequence of inner loops followed by the presentation of a context cue. We simulated the HER model on a version of the 1-2AX task as described in O'Reilly & Frank (2006)^3^ in which each outer loop consisted of 1-4 inner loops, and the probability of observing a valid sequence was 0.25. There were 8 inputs to the model, corresponding to the 6 relevant cues in the task, as well as 2 distractor cues that had no task relevance. At each cue, the model made a response to indicate whether the current stimulus was a target or not. In order to perform the task correctly, target responses should be made only at the presentation of a valid target cue; all other cues should result in non-target responses. Feedback to the model indicated correct or incorrect performance. We simulated the 1-2AX task on approximately 24,000 individual cue presentations as described in previous work^1^. The activity of each prediction unit at each higher level was recorded on the presentation of a potential target cue ('X' or 'Y') to be used as input to a 2 level feedforward neural network with 10 hidden units. The neural network was trained on the sequence of high ('1' & '2') and low ('A' & 'B') level context cues using the MATLAB^13^ neural networks toolbox.

*Simulation 3:Miller, Erickson, and Desimone (1996)*^14^

The model was simulated for 6000 trials on a simple delayed match-to-sample (DMTS) task. On each trial, a neutral stimulus indicating the beginning of a trial was presented, followed by one of two sample stimuli, and ending with one of two target stimuli. There were a total of 3 inputs to the model, 1 indicating the trial onset, and 2 for the task-relevant stimuli. Note that sample stimuli and target stimuli used the same representation. The model could make two responses indicating either a match or non-match between the sample and target stimuli, and each response resulted in either correct or incorrect feedback, for a total of 4 outcome units.

*Simulation 4:Wallis, Anderson & Miller, 2001*^15^

The model was simulated for 6000 trials on a version of the DMTS task. As in simulation 3, the model was presented with one of two sample stimuli, and one of two possible target stimuli. Prior to presentation of either stimulus, one of four rule cues were presented. Two of the rule cues (MATCH) indicated that the model should make a target response if the sample and target stimuli matched, and a non-target response otherwise, while the other two rule cues (NON-MATCH) indicated that the model should make a target response to mismatching sample/target cues. Each rule (MATCH and NON-MATCH) had two, non-overlapping representations in the model, reflecting the different modalities used to cue rules in Wallis et al., 2001 ^15^.

*Simulation 5: Markant & Gureckis (2012)*^16^

The model was simulated on a ternary probability estimation task for 5000 trials in three different learning conditions. Task stimuli were modeled as compound stimuli composed of two feature dimensions, and each dimension had three possible values as described in previous work^1^. Each unique conjunction of feature dimension values was associated with one of three possible responses such that each feature of each dimension was associated with each of the three possible responses in only one instance. The learning rate of the model was manipulated across the three learning conditions as follows: in the no learning condition, the learning rate for all hierarchical levels of the model was set to 0, and thus no learning occurred during the experiment. In a second learning condition, learning was enabled only for the lowest hierarchical level and set to the parameter values reported in table 1. Finally, in the third learning condition, learning was enabled for all levels, and set to the parameter values in table 1.

*Simulation 6: Kim et al. (2011)*^17^

In Kim et al. (2011), the authors attempted to identify brain activity related to set switches at various levels of abstraction. Subjects were presented with a colored (red or green) box situated in a single cell of a 2X2 grid. The color of the box indicated a cognitive set of two numbers that the box might indicate; a red box, for instance, may indicate either 5 or 7, depending on the box's horizontal position (left/right columns). A red box appearing in the left column may indicate a 5, while a red box in the right column indicates a 7. The vertical position of the box (upper/lower rows) indicated which of two operations (greater than/less than) the subject should perform in comparing the number indicated by the position and color of the box with a plain digit presented alongside the box. Changes in the position and color of the box indicate set switches at various levels of abstraction: changes in the left/right position indicate stimulus switches, changes in the upper/lower position indicate switches in responses, and changes in box color indicate cognitive set switches. The model was simulated on 20,000 trials, and activity was calculated from the final 2000 trials. A total of 6 inputs were modeled, reflecting the 2 colors, 2 horizontal positions, and 2 vertical positions possible in the task.

*Simulation 7: Gehring & Knight (2000)*^18^

The model was simulated for 6000 trials on a simple delayed match-to-sample (DMTS) task. On each trial, a neutral stimulus indicating the beginning of a trial was presented, followed by one of two sample stimuli, and ending with one of two target stimuli. There were a total of 3 inputs to the model, 1 indicating the trial onset, and 2 for the task-relevant stimuli. Note that sample stimuli and target stimuli used the same representation. The model could make two responses indicating either a match or nonmatch between the sample and target stimuli, and each response resulted in either correct or incorrect feedback, for a total of 4 outcome units. Two conditions were simulated: a control condition in which all pathways were intact, and a lesion condition in which the value of P' in Eq. 8 was set to 0, effectively removing any top-down influence between levels. Activity was recorded from all 6000 trials.

|  |  |  | **Value for:** | | |
| --- | --- | --- | --- | --- | --- |
| **Parameter** | **Description** | **Equation** | **Layer 1** | **Layer 2** | **Layer 3** |
| $\alpha$ | Learning Rate | 3 | 0.1 | 0.02 | 0.02 |
| $\lambda$ | Eligibility Trace Decay | 5 | 0.3 | 0.5 | 0.9 |
| $\beta$ | Working Memory Update Gain | 6 | 12 | 12 | 12 |
| $\gamma$ | Response Selection Gain | 10 | 12 | N/A | N/A |

Table 1: Parameter set for all simulations

**Supplementary Results**

In the main text, we discuss seven sets of simulations selected to illustrate the breadth of results that can be accommodated by the HER model. While the studies highlighted in the main text are useful for describing the principles and mechanisms by which the HER model operates and is able to capture empirical results related to neural activity and behavior, the model was developed with the goal of providing a unifying framework for interpreting a broad range of results concerning the function of mPFC and dlPFC. Accordingly, we include results of additional simulations, described below, based on published studies in this section in order to demonstrate the range of findings that can be captured by the HER model under various experimental manipulations. Simulations reported here used the same parameter set as those reported in the main text, and the model learned each simulated task autonomously.

*Reynolds et al., 2012*

We refer readers to the original paper for a complete description of the task described in Reynolds et al. (2012)^19^. Briefly, subjects performed a task in which both the level of control/abstraction and WM maintenance duration were manipulated. Subjects experienced 5 conditions, a baseline condition with low control and no maintenance requirements, as well as 4 conditions reflecting two levels of control (high and low) crossed with two levels of maintenance requirements (single- and multi-trial). Conditions were presented as a block of 5 trials, each of which consisted of 3 stimuli presented sequentially. For the 1st and 2nd stimuli presented within a trial, a default response was made. Upon presentation of the 3rd stimulus, the subject was required to make a response which may or may not have been contingent on the preceding stimulus or stimuli. We simulated each of these conditions using a separate instantiation of the HER model. The model was simulated for 3000 blocks (1 block = 5 trials) for each condition, and activity was calculated using only the last 100 blocks. The 21 Inputs to the model corresponded with those reported in Reynolds et al (2012)^19^, and included 4 color inputs, 6 number cues, 10 letter cues, and a null cue which had no influence on responses. The model could generate 3 responses, a default response indicating a non-response, and two target responses. Feedback informed the model of correct or incorrect outcomes following each response.

In Reynolds et al. (2012)^19^, the authors attempt to distinguish between two competing accounts of dlPFC activity, the Information Cascade account^11^ and the Level of Abstraction hypothesis ^20^, which states that progressively more abstract stimuli activate regions of dlPFC along the rostrocaudal abstraction gradient. To do so, they manipulated both the degree of abstraction indicated by a stimulus as well as the maintenance demands in terms of the duration for which an informative stimulus should be maintained. The authors found that neither account satisfactorily explained the observed data, and instead proposed the Adaptive Context Maintenance hypothesis which states that the temporal dynamics of dlPFC change in response to shifts in task demands. The authors report data consistent with this hypothesis in which the relative contribution of two sources of dlPFC activity, sustained and transient activation, shifts with maintenance demands. The HER model is able to account for this pattern (Figure S2) through learned WM gating weights: for conditions in which items must be maintained for short durations, model activity related to transient updates is greater than for maintenance activity (see Online Methods; eqs 13 and 14) as items are encoded and purged from WM with greater frequency, while the reverse is true in which items must be maintained over longer periods.

Additionally, the model is able to capture the overall pattern of data the authors report in mid dlPFC. Model activity (see Online Methods; eq. 16) at level 2 increases both as the level of contextual control increases as well as maintenance demands increase (fig S2A). The increase in activity with contextual control is explained as follows: as contextual control increases, so too does the strength of error predictions maintained in the HER model. This is intuitively appealing, insofar as conditions in which increased control is mandated are exactly those conditions in which errors are more likely, and thus error predictions are stronger.

**
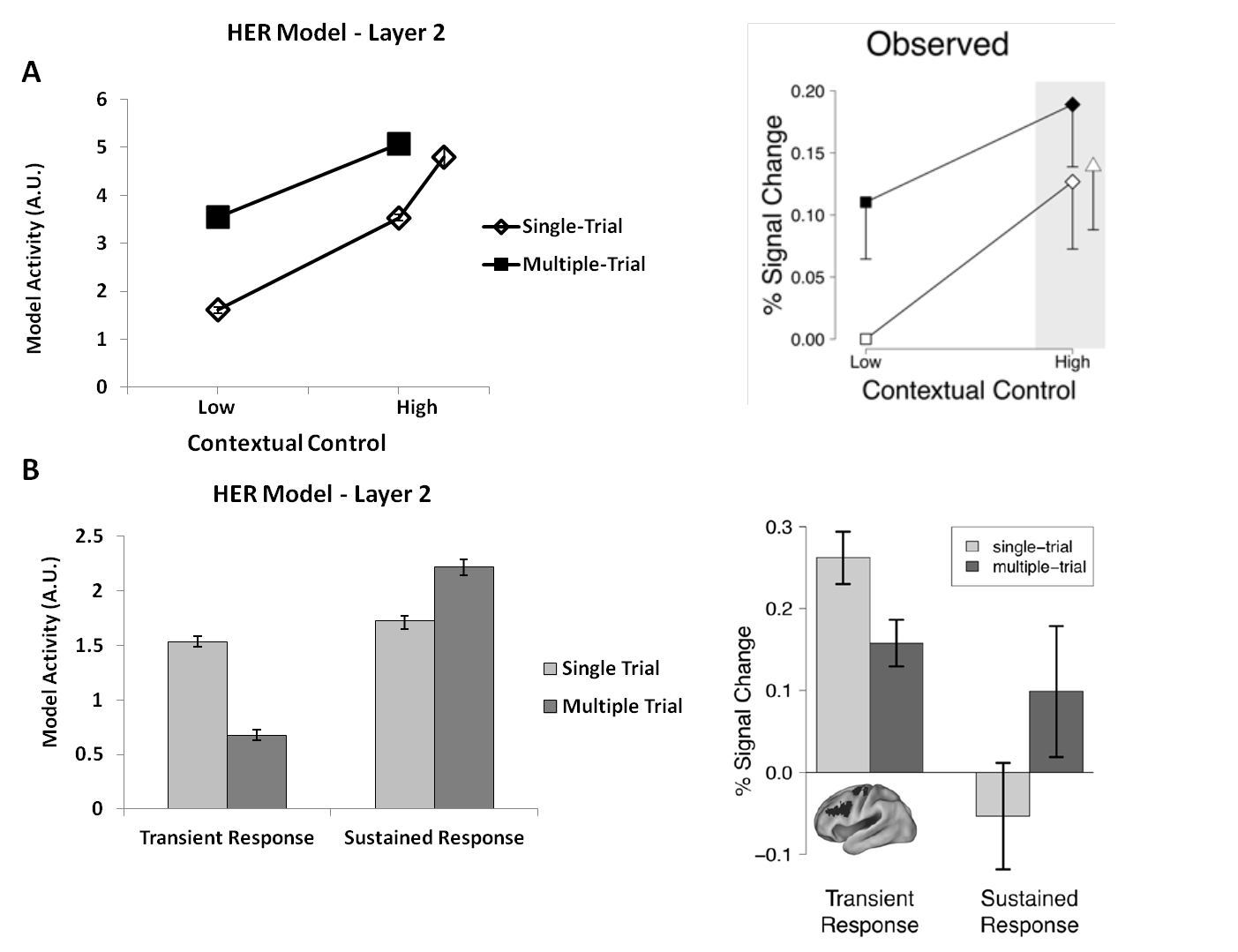
**

Figure S2: Reynolds et al., 2012. A) Level 2 of the HER model captures the pattern of increasing activity observed in mid-dlPFC as the degree of contextual control (x-axis) and maintenance demands (separate lines) increases. B) The relative contributions of transient and sustained components of activity in the model (cf. eqs 13 & 14 in Online Methods) adjust to maintenance demands. Transient signals in Level 2 of the model contribute more to model activity under short-maintenance conditions, while sustained signals are more pronounced in long-maintenance conditions. This pattern is observed in mid-dlPFC in human subjects.

*Nee , Jahn and Brown, 2013*

In Nee, Jahn & Brown (2013)^21^, the authors manipulated two forms of abstraction: temporal and relational. Two cues were presented sequentially, informing subjects which concrete feature of an imminent compound stimulus should be attended, and which of three logical operations (OR, AND, XOR) should be performed on that feature. Temporal abstraction was manipulated by the order in which the cues associated with concrete features and logical operators were presented. The authors observed regions in rostral and caudal dlPFC whose activity corresponded to cues indicating logical operations rather than features, while regions in caudal SFS showed no differences. The HER model captures the pattern of activity observed across regions of dlPFC (rostral, caudal, and SFS) at the presentation of the first and second cue in both temporal abstraction conditions (fig S3). Interestingly, although the authors report that rostral dlPFC as well as caudal dlPFC represent relational abstraction (i.e., increased activity at the onset of a cue indicating which logical operation to perform regardless of the order in which it is presented), the HER model learns to map the relational cue to the highest hierarchical level, while the middle hierarchical level preferentially encodes the concrete feature.

Additionally, the HER model predicts that activity at the lowest level, nominally associated with PMd, should be equivalent in each of the temporal abstraction conditions. Compare this to the predictions of the HER model in the Koechlin et al., 2003^11^ Task Experiment (see Main Text) in which the model predicts equivalent activity regardless the amount of contextual information conveyed by a cue. One possible reason the model fails with regard to the Koechlin et al. prediction is that the region identified in that study as PMd extends into regions other authors have labeled as “pre-PMd”^19^. It may therefore be the case that the pattern of activity reported by Koechlin et al. for PMd is composed of a mixture of signals from adjacent hierarchical levels. More generally, we note that the HER model consistently reproduces the posterior-to-anterior gradient of increasing abstraction, but the particular brain regions that represent corresponding levels of abstraction may vary even though the overall posterior-to-anterior abstraction gradient structure is preserved.


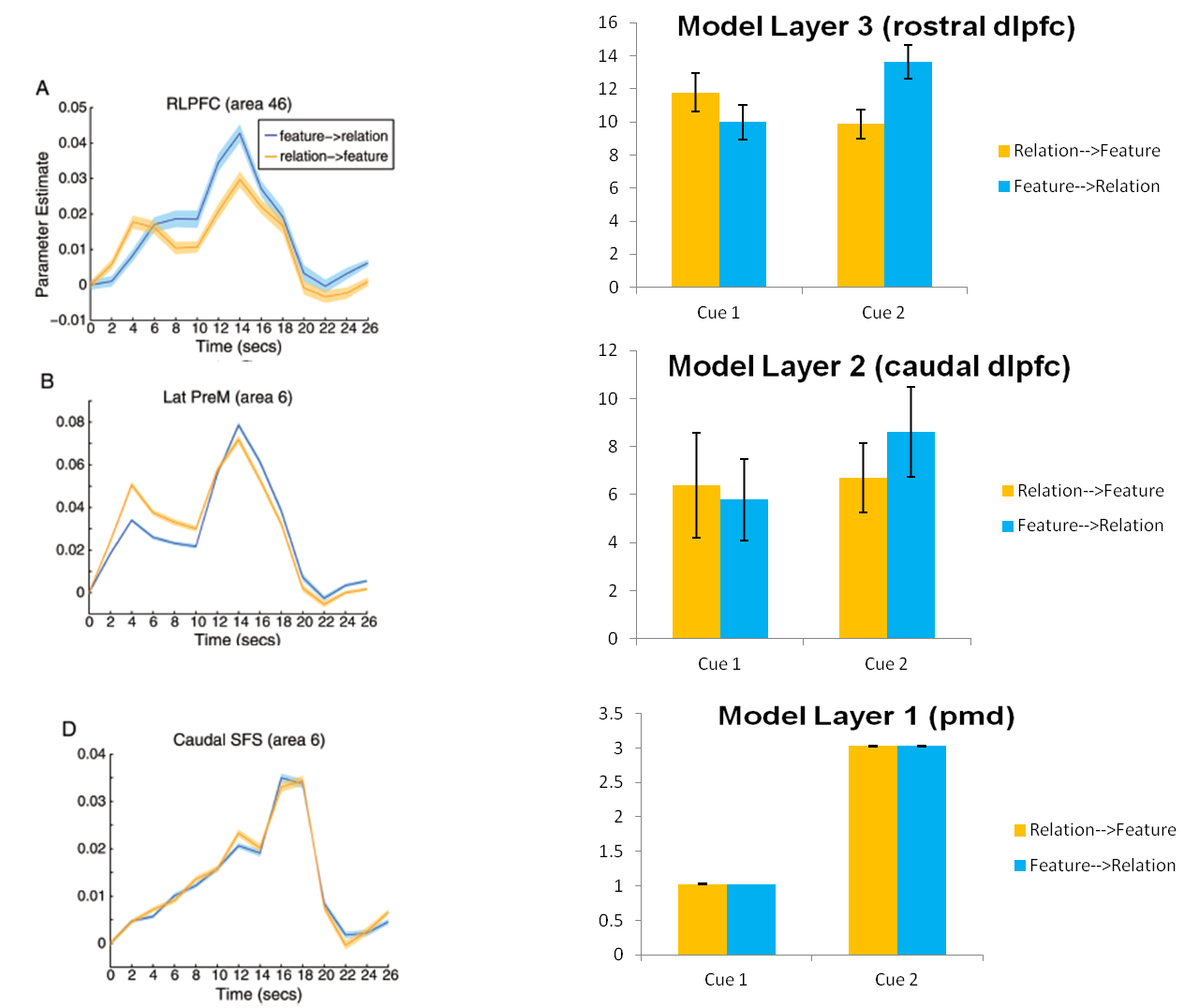


**C**

Figure S3: *Nee, Jahn, & Brown, 2014*. Left Frames: Activity in rostral (A) and caudal (B) dlPFC is sensitive to manipulations of the order in which a concrete (feature) and abstract (relation) cue is presented. Activity for both regions increases for on presentation of the relation cue relative to the feature cue regardless whether it is presented first (0 seconds) or second (10 seconds). Activity in premotor regions (C) shows no differences in activity related to the order of presentation. Right Frames: The HER model captures the overall effect of increased activity in both Level 3 and Level 2 on presentation of the relation cue, as well as the equivalence of activity in PMd regardless of order. In contrast to the interpretation offered in the original study, however, the HER model learns to preferentially map relation cues to highest level of the model, and feature cues to level 2.

*Nee and D’Esposito, 2016*

In Nee & D’Esposito (2016)^22^, subjects engaged in a sequence matching task in which factors of contextualization and integration were manipulated. In a control condition, subjects were asked to respond whether a currently observed stimulus sequentially followed a previously presented stimulus. In a *restart* condition, subjects performed the basic control task, but midway through a block were instructed, via a change in the identity of a visual cue, to perform the task as if they were beginning a new block. In a *delay* condition, the change in identity of the visual cue instructed subjects that they were to maintain the last stimulus observed in working memory while a number of distractors were presented. Following the reversion of the visual cue to the original identity, subjects continued performing the task. A fourth condition, *dual,* was performed by subjects, but is not included in the simulations reported here. The model was simulated for 1500 blocks (1 block = 24 trials), and model activity was recorded for each trial. A total of 11 inputs were modeled: 1 input indicated the onset of a new block, while 10 inputs indicated the combination of sequence position and stimulus identity. Model responses consisted of a target and non-target response.


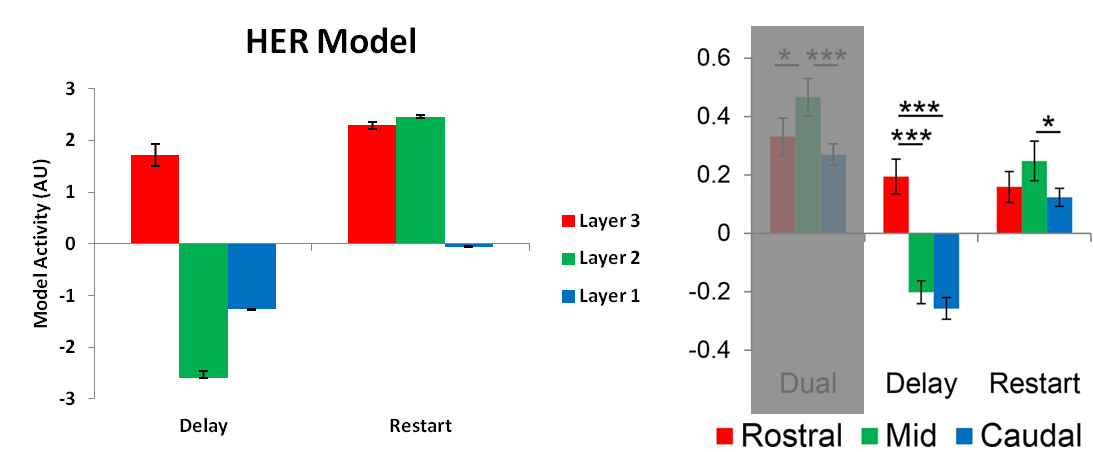


**B)**

**A)**

Figure S4: *Nee & D'Esposito, 2016*. (A) Activity in the 3 levels of the HER model qualitatively matches (B) the activity observed in subjects performing a sequence recognition task under 2 experimental manipulations. Activity in Level 1 (blue bars) is higher for restart and control conditions than for delay conditions. Activity in Level 2 (red) is highest for restart conditions and lowest for delay conditions. Activity in Level 3 (green) is highest in the delay and restart conditions relative to the control conditions.

Again, the HER model captures the pattern of activity across conditions for three regions of lPFC, corresponding to the three hierarchical levels used in our simulations (Figure S4). At the lowest level, model activity is higher for *restart* and control conditions relative to the delay condition. In the middle hierarchical level, activity is highest for the *restart* condition, while at the highest level, activity is increased for the *delay* and *restart* condition relative to the control condition. The HER model is able to reproduce this pattern due to the functional roles learned by each level: the lowest level, roughly equivalent to PMd, learns to respond to local relationships – subsequent items in a sequence – and thus the increased activity in the *restart* and control conditions is a result of trial-by-trial updates of anticipated responses, while being relatively unaffected by delays. Activity in the middle level – caudal dlPFC – reflects the update and maintenance of currently valid sequences in the *restart* condition, while being relatively uninfluenced by delays or trial-to-trial updates. Final, the top level – rostral dlPFC – is involved primarily in maintaining information across delays, and thus activity is higher for delay and *restart* conditions in which information must be maintained over periods of time, or the identity of the currently valid sequence needs to be maintained.

**Supplementary Discussion**

The above simulations demonstrate the HER model's ability to account for a range of additional data from fMRI beyond those simulations included in the main text. Notably, the simulations described above used an identical parameter set as those in the main text, and learned the tasks described in the respective studies in an autonomous and self-organized fashion. This is of particular interest insofar as the tasks described in many of the simulated studies reflect a variety of approaches to investigating sophisticated processes that are considered to be a hallmark of human cognition.

The HER model somewhat incidentally provides a solution to the problem of how working memory is stored, maintained, and released ^23^. The HER model learns to store incoming stimuli as items in working memory specifically when those items correlate with an upcoming prediction error. In that case, error signals train the WM to store anything that correlates with prediction errors, for as long as the items correlate with a likely prediction error; afterwards they are released or overwritten. These WM representations are then available to guide behavioral responses. In this way, the HER model simulates how proactive control might be provided, as items with high information content are stored in WM to then guide responses to subsequent target stimuli.

The HER model assumes that each layer in the model stores a single item. This is consistent with empirical findings and models of limited capacity ^24,25^. While the model is only able to maintain a single item in WM at each layer, each item is associated with more complicated error representations that modulate the function of inferior layers, and are themselves modulated by the activity of superior layers. The model thus has a greater expressive capacity than may be indicated by the representation of single items. Additionally, the ability of the model to effectively gate appropriate representations into WM at each point in time is influenced by softmax gain parameters (eq. 6). Previous investigation of the influence of hyperparameters on model performance^1^ found that model performance (trials to criterion) was optimal when the gain parameter at each hierarchical level followed a gradient, potentially corresponding to patterns of dopamine innervation of PFC observed in monkey^26^. This constitutes a novel prediction of the model, suggesting that differences in patterns of dopamine innervation of hierarchical PFC regions may correspond to differences in performance and learning. The softmax gain parameter/precision has likewise been associated with dopamine function in the context of predictive coding and active inference^7^.

The HER model assumes a rigid, layer-wise structure in which each hierarchical level in the model is associated with a specific region in PFC corresponding to a putative rostrocaudal abstraction gradient. There are various reasons to suppose that this assumption may not be completely correct. Hierarchical organization in PFC is thought to be supported by overlapping cortico-ganglia loops ^27^ that may play a critical role in gating information into ^3^ and out of ^28^ WM. This overlap of information gating suggests that the information represented in various subregions of PFC may not correspond to a "pure" level of abstraction, but a probabilistic mix of more concrete and more abstract signals. Additionally, the assumption that rostral regions of PFC correspond to the apex of the abstraction gradient have recently been questioned by work examining anatomical data from monkey^29^ and human fMRI data ^30^, suggesting that the peak of the hierarchical gradient might lie caudal to rostral dlPFC. While additional evidence is needed, one possibility is that the hierarchical organization suggested by the HER model applies in the specific type of task (discussed below) the model was developed to perform. Additional task types, e.g., prospective planning, may imply a distinct hierarchical organization.

Although the HER model accounts for a wide range of effects observed in mPFC and dlPFC, these effects derive from tasks in which previously observed information must be integrated in order to govern responses to a current stimulus. This represents one operating mode of the cognitive control network that is, in a sense, primarily reactive – the network responds to information that has been observed to maximize the likelihood of correct performance, but cannot itself select the information that will be observed in the future. An alternate mode, not captured by the HER model, may be regarded as primarily proactive – given a current state, how should a network behave in order to realize a desired goal? Put another way, this conception of proactive control requires the network to select future states that need to be observed in order to achieve a goal. Although the HER model was not developed with the intent of capturing this form of control, it is possible that goal-oriented behavior of this sort might be supported by an architecture similar to the HER model. While additional work is needed to explore this question, it suggests a plausible interpretation of how goals might be represented in the brain. Specifically, since the HER model is based on a predictive coding framework, high-level goals may be interpreted as deviations between a current state and a desired state that inform responses to a current stimulus.

**References:**

1. Alexander, W. H. & Brown, J. W. Hierarchical Error Representation: A Computational Model of Anterior Cingulate and Dorsolateral Prefrontal Cortex. *Neural Computation* 1–57 (2015). doi:10.1162/NECO_a_00779

2. Alexander, W. H. & Brown, J. W. Medial prefrontal cortex as an action-outcome predictor. *Nat Neurosci* **14,** 1338–1344 (2011).

3. O’Reilly, R. C. & Frank, M. J. Making working memory work: a computational model of learning in the prefrontal cortex and basal ganglia. *Neural computation* **18,** 283–328 (2006).

4. Hazy, T. E., Frank, M. J. & O’Reilly, R. C. Banishing the homunculus: making working memory work. *Neuroscience* **139,** 105–18 (2006).

5. O’Reilly, R. C., Frank, M. J., Hazy, T. E. & Watz, B. PVLV: the primary value and learned value Pavlovian learning algorithm. *Behavioral neuroscience* **121,** 31–49 (2007).

6. Friston, K. The free-energy principle: a rough guide to the brain? *Trends in Cognitive Sciences* **13,** 293–301 (2009).

7. Friston, K., FitzGerald, T., Rigoli, F., Schwartenbeck, P. & Pezzulo, G. Active Inference: A Process Theory. *Neural Computation* **29,** 1–49 (2016).

8. Alexander, W. H. & Brown, J. W. A general role for medial prefrontal cortex in event prediction. *Front. Comput. Neurosci.* **8,** 69 (2014).

9. Riggall, A. C. & Postle, B. R. The Relationship between Working Memory Storage and Elevated Activity as Measured with Functional Magnetic Resonance Imaging. *J. Neurosci.* **32,** 12990–12998 (2012).

10. Lundqvist, M. *et al.* Gamma and Beta Bursts Underlie Working Memory. *Neuron* **90,** 152–164 (2016).

11. Koechlin, E., Ody, C. & Kouneiher, F. The architecture of cognitive control in the human prefrontal cortex. *Science* **302,** 1181–5 (2003).

12. Nee, D. E. & Brown, J. W. Dissociable frontal-striatal and frontal-parietal networks involved in updating hierarchical contexts in working memory. *Cereb. Cortex* **23,** 2146–2158 (2013).

13. The MathWorks, Inc. *MATLAB 2012b*.

14. Miller, E. K., Erickson, C. A. & Desimone, R. Neural mechanisms of visual working memory in prefrontal cortex of the macaque. *Journal of neuroscience* **16,** 5154–5167 (1996).

15. Wallis, J. D., Anderson, K. C. & Miller, E. K. Single neurons in prefrontal cortex encode abstract rules. *Nature* **411,** 953–956 (2001).

16. Markant, D. & Gureckis, T. One piece at a time: Learning complex rules through self-directed sampling. in *Proceedings of the 34th Annual Conference of the Cognitive Science Society* 725–730 (Cognitive Science Society, 2012).

17. Kim, C., Johnson, N. F., Cilles, S. E. & Gold, B. T. Common and Distinct Mechanisms of Cognitive Flexibility in Prefrontal Cortex. *J. Neurosci.* **31,** 4771–4779 (2011).

18. Gehring, W. J. & Knight, R. T. Prefrontal-cingulate interactions in action monitoring. *Nat Neurosci* **3,** 516–520 (2000).

19. Reynolds, J. R., O’Reilly, R. C., Cohen, J. D. & Braver, T. S. The function and organization of lateral prefrontal cortex: a test of competing hypotheses. *PLoS One* **7,** e30284 (2012).

20. Badre, D. Cognitive control, hierarchy, and the rostro-caudal organization of the frontal lobes. *Trends Cogn. Sci. (Regul. Ed.)* **12,** 193–200 (2008).

21. Nee, D. E., Jahn, A. & Brown, J. W. Prefrontal Cortex Organization: Dissociating Effects of Temporal Abstraction, Relational Abstraction, and Integration with fMRI. *Cereb. Cortex* bht091 (2013). doi:10.1093/cercor/bht091

22. Nee, D. E. & D’Esposito, M. The hierarchical organization of the lateral prefrontal cortex. *eLife* **5,** e12112 (2016).

23. Badre, D. & Frank, M. J. Mechanisms of Hierarchical Reinforcement Learning in Cortico–Striatal Circuits 2: Evidence from fMRI. *Cereb. Cortex* **22,** 527–536 (2012).

24. Nee, D. E. & Jonides, J. Trisecting representational states in short-term memory. *Front Hum Neurosci* **7,** (2013).

25. Frank, M. J. & Badre, D. Mechanisms of Hierarchical Reinforcement Learning in Corticostriatal Circuits 1: Computational Analysis. *Cereb. Cortex* **22,** 509–526 (2012).

26. Williams, S. M. & Goldman-Rakic, P. S. Characterization of the dopaminergic innervation of the primate frontal cortex using a dopamine-specific antibody. *Cereb. Cortex* **3,** 199–222 (1993).

27. Draganski, B. *et al.* Evidence for Segregated and Integrative Connectivity Patterns in the Human Basal Ganglia. *J. Neurosci.* **28,** 7143–7152 (2008).

28. Chatham, C. H., Frank, M. J. & Badre, D. Corticostriatal output gating during selection from working memory. *Neuron* **81,** 930–942 (2014).

29. Goulas, A., Uylings, H. B. M. & Stiers, P. Mapping the Hierarchical Layout of the Structural Network of the Macaque Prefrontal Cortex. *Cereb Cortex* **24,** 1178–1194 (2014).

30. Nee, D. E. & D’Esposito, M. The hierarchical organization of the lateral prefrontal cortex. *eLife Sciences* **5,** e12112 (2016).
